# Differences in Blood Pressure Levels Among Children by Sociodemographic Status

**DOI:** 10.5888/pcd18.210058

**Published:** 2021-09-16

**Authors:** Melissa Goulding, Robert Goldberg, Stephenie C. Lemon

**Affiliations:** 1 Division of Preventive and Behavioral Medicine, Department of Population and Quantitative Health Sciences, University of Massachusetts Medical School, Worcester, Massachusetts; 2 Division of Epidemiology, Department of Population and Quantitative Health Sciences, University of Massachusetts Medical School, Worcester, Massachusetts

## Abstract

**Introduction:**

The American Academy of Pediatrics (AAP) updated its blood pressure (BP) screening guidelines in 2017 to emphasize body weight as a risk factor. We provide contemporary, nationally representative estimates of prevalence of elevated and hypertensive BP among US children and examine sociodemographic prevalence differences, accounting for the influence of weight.

**Methods:**

We used cross-sectional data from children aged 8 to 17 years (N = 5,971; weighted N = 36,612,323) collected from 2011 through 2018 in 4 biennial cycles of the National Health and Nutrition Examination Survey (NHANES). Children’s BP was categorized as normal, elevated, or hypertensive. Sociodemographic characteristics included were sex, age, race/ethnicity, family income, and education. Log binomial regression, with and without adjustment for weight (dichotomized at the 85th body mass index percentile), determined prevalence estimates and differences for elevated and hypertensive BPs with 95% CIs.

**Results:**

In NHANES data collected from 2011 through 2018, 7.2% (95% CI, 6.3%–8.3%) of US children had elevated BP, and 3.8% (95% CI, 3.3%–4.5%) had hypertensive BP according to 2017 AAP guidelines. Differences in prevalence of weight-adjusted elevated BP indicated higher prevalence among children aged 16 to 17 years compared with children aged 8 to 9 years (prevalence difference, +6.3%; 95% CI, 3.2%–9.4%), among males compared with females (+4.6%; 95% CI, 2.7%–6.4%), and among non-Latino Black children compared with non-Latino White children (+4.0%; 95% CI, 2.2%–5.8%). Crude hypertensive BP prevalence was highest among children aged 8 to 9 years, male children, and Mexican American children. The only difference remaining after weight adjustment was among children aged 8 to 9 years and 13 to 15 years.

**Conclusion:**

Elevated BP was most prevalent among US children who were older, male, or non-Latino Black. Factors beyond inequalities in body weight may contribute to disparities in elevated BP.

**Summary**

**What is already known on this topic?**

High blood pressure (BP) affects many US children; however, most prevalence estimates are based on outdated data and guidelines. Although studies have shown that childhood hypertensive BP is not evenly distributed across sociodemographic groups, they do not account for body weight as a contributor to prevalence disparities.

**What is added by this report?**

Our study provides contemporary national prevalence estimates of elevated and hypertensive BP among children across sociodemographic groups and examines the effect of weight on observed disparities.

**What are the implications for public health practice?**

Factors beyond inequalities in body weight may contribute to disparities in elevated BP among US children. Further investigation of these disparities is needed to inform targeted public health efforts.

## Introduction

Hypertension affected nearly 4% of US children from 2013 through 2016 (1). The high prevalence of childhood obesity has contributed to an increase in several chronic conditions among children, including hypertension (2). Children who are overweight have higher systolic and diastolic blood pressure (BP) (3) than normal-weight children, and those with obesity have a threefold higher risk of hypertension compared with children of healthy weight (4). Given the relationship between weight and BP, the American Academy of Pediatrics (AAP) changed its clinical practice guidelines in 2017 with new normative pediatric BP tables to assess children’s BP percentiles and categories on the basis of healthy body weight, in contrast to their previous guidelines, which included children of all weight statuses (5). Prevalence estimates based on AAP’s earlier guidelines may have been biased by body weight and therefore warrant reinvestigation. Although AAP’s guideline changes increased estimated prevalence of hypertension among US children (from 1.9% to 3.5%) (1), national estimates beyond 2016 are unavailable (1,5,6).

Few studies have described sociodemographic factors associated with hypertension among US children. Although prevalence in those studies appears to be higher among males and among Black, Mexican American, and other Latino children (1,5,7–9), many of those studies were based on past AAP guidelines (10) and few investigated the extent to which disparities in BP could be explained by differences in weight (7,9). Furthermore, investigation of potential associations between hypertension and socioeconomic factors has been limited (11,12).

The objective of our study was to provide nationally representative prevalence estimates of elevated and hypertensive BP among US children according to 2017 AAP guidelines. We also examined sociodemographic differences in prevalence and explored the role of weight status in relationship to differences in BP levels.

## Methods

### Study design and database

Our cross-sectional study used nationally representative data from the National Health and Nutrition Examination Survey (NHANES) (13), which is collected biennially by the National Center for Health Statistics to provide data on the health status of community-dwelling US residents. NHANES collects sociodemographic, dietary, and general health information by survey and medical, dental, and laboratory data by physical examination. We used data from 2011–2018, which consists of 4 biennial cycles. Unweighted survey response rates ranged from 53.6% to 78.5% for our study sample. Additional adjustments to weighting procedures were used to reduce the potential effects of response bias resulting from a lower response rate in the 2017–2018 NHANES cycle (13). NHANES data collection is approved by the National Center for Health Statistics Research and Ethics Review Board. Participant and parental consent were obtained for children aged 13 years or older. Participant assent and parental consent were obtained for children aged 7 to 12 years.

### Study population

NHANES BP data comes from physical examinations (13). For our study we included children aged 8 to 17 years for whom data on BP, height, weight, race/ethnicity, and socioeconomic characteristics were available. We excluded children who were missing BP measurements (n = 338), had fewer than 3 BP readings (n = 68), were missing data on body mass index (BMI) (weight in kg/height in m^2^) (n = 32), or were missing data on sociodemographic characteristics (n = 702). The final sample included 5,971 children, weighted to represent 36,612,323 children. To provide biennial prevalence estimates of hypertensive and elevated BP, the sample was defined by NHANES cycle. We used the entire sample for prevalence estimates of various BP parameters and differences in these end points according to sociodemographic factors.

**Operational definition of pediatric elevated and hypertensive BP**. Although clinical diagnosis of hypertension requires BP measurement across at least 3 occasions, NHANES is limited to physical examination on 1 occasion. Therefore, 3 BP measurements taken on a single occasion were averaged for each child in accordance with AAP guidelines for clinicians and common practice in pediatric hypertension studies (1,5,7–9). NHANES BP measurement techniques have been described previously (13). For children aged 8 to 12 years, we used age, sex, and height to determine their BP percentile according to the 2017 AAP BP tables. BP percentiles (for children aged <13 y) or average measurement (for children aged 13–17 y) were then used for categorization according to 2017 AAP guidelines. Elevated BP was defined as ranging from ≥90th percentile to <95th percentile or 120/<80 mm Hg to <95th percentile (whichever is lower) for children aged 8 to 12 years and 120/<80 to 129/<80 mm Hg for those aged 13 to 17 years. Hypertensive BP was defined as a BP percentile of ≥95 or an average BP of ≥130/80 mm Hg (whichever was lower) for children aged 8 to 12 years and ≥130/80 mm Hg for those aged 13 to 17 years.

**Body mass index percentile**. Children’s standing height and weight were measured by trained professionals during the NHANES physical examination, and their BMI was calculated. Methods and equipment used for anthropometric measures have been described previously (14). We determined BMI percentiles according to the Centers for Disease Control and Prevention 2000 growth charts (15). Weight status was categorized by BMI percentile to represent healthy weight (BMI percentile <85), overweight (BMI percentile ≥85 to <95), and obesity (BMI percentile ≥95). For adjusted prevalence estimates, we dichotomized weight to indicate unhealthy weight status (BMI percentile ≥85).

**Sociodemographic factors associated with elevated and hypertensive BP.** Age at the time of the NHANES physical examination was determined by the child’s date of birth and was stratified at 8 to 9 years, 10 to 12 years, 13 to 15 years, and 16 to 17 years. Sex was determined by self-report with options of male or female. We used the more inclusive NHANES race/ethnicity variable in which children who identified as Mexican American were coded as such, those who identified as Hispanic or Latino were coded as other Latino, and those who identified as non-Latino were coded according to self-reported race of White, Black, Asian, or other (American Indian or Alaska Native, Native Hawaiian or Pacific Islander, mixed race).

We used 2 proxy measures for socioeconomic status, family poverty income ratio (PIR) and parent/guardian education level. PIR was calculated by dividing family income by the Department of Health and Human Services’ poverty guidelines and then categorized as low (PIR <1.3), medium (PIR ≥1.3 and <3.5), and high (PIR ≥3.5). This categorization was used to be consistent with past obesity-related research and because a PIR of <1.3 is often used to determine eligibility for federally funded programs, including the Supplemental Nutrition Assistance Program (16). Parent/guardian education level was measured as the highest education of the household reference person, who was the first person listed in the household aged 18 years or older who owned or rented the residence.

### Statistical analysis

We computed frequencies on our study sample. Because each of the continuous variables had nonnormal distributions (assessed via Shapiro–Wilk test), medians with interquartile range were calculated. Prevalence estimates of elevated and hypertensive BP were computed for the 2011–2018 period overall and by 4 biennial cycles. We estimated crude prevalence differences and weight status (BMI percentile ≥85) adjusted prevalence differences with 95% CIs for elevated and hypertensive BP for each sociodemographic subgroup through log binomial regression with the identity link (17). Each sociodemographic factor was assessed separately. Models were then adjusted for weight status. Assessment of correlations between weight status and each sociodemographic variable suggested adjusted models were not collinear. All analyses were appropriately weighted and analyzed with examination sample weights and Taylor series linearization (13) accounting for the complex sampling design of NHANES.

## Results

**Characteristics of US children**. Among children aged 8 to 17 years in NHANES 2011–2018, nearly a third (31.1%) were aged 13 to 15 years (Table 1). About half were female (49.7%). More than half (55.4%) were non-Latino White. The next largest racial/ethnic group was Mexican American (14.1%). Over one-third (37.6%) had an unhealthy body weight index (BMI) (≥85 percentile).

**Table 1:** Characteristics of Noninstitutionalized US Children Aged 8 to 17 Years, National Health and Nutrition Examination Survey (NHANES) 2011–2018

Characteristic	Children (Unweighted, N = 5,971; Weighted, N = 36,612,323)^a^
**Age, y**	
8–9	18.9
10–12	29.2
13–15	31.1
16–17	20.9
**Female**	49.7
**Race/ethnicity**	
Non-Latino White	55.4
Non-Latino Black	13.8
Mexican American	14.1
Other Latino	7.1
Non-Latino Asian	4.1
Other^b^	5.5
**Highest level parent/guardian education**	
≥College graduate	28.8
High school diploma/GED/some college	52.6
<High school diploma	18.5
**Family income^c^**	
High	30.1
Medium	39.2
Low	30.7
**Weight status**	
BMI percentile, median (IQR)	73.3 (42.7-93.0)
Healthy weight (BMI percentile <85)	62.5
Overweight (BMI percentile ≥85 to <95)	17.0
Obesity (BMI percentile ≥95)	20.6

Abbreviation: BMI, body mass index; IQR, interquartile range.

a Values are weighted percentage unless otherwise indicated.

b Includes American Indian or Alaska Native, Native Hawaiian or Pacific Islander, and mixed race.

c Determined by family poverty income ratio (PIR): family income divided by Department of Health and Human Services poverty guidelines (specific to family size, year, and state of residence). High = PIR >3.5, medium = PIR ≥1.3, < 3.5; low = PIR <1.3.

**Prevalence of elevated and hypertensive BP**. In the most recent NHANES cycle, 2017–2018, the prevalence of elevated BP was 6.2% (95% CI, 4.2%–9.3%) (Table 2) and the prevalence of hypertensive BP was 3.9% (95% CI, 2.9%–5.3%). Prevalence of hypertensive BP overall from 2011–2018 was 3.8% (95% CI, 3.3%–4.5%).

**Table 2:** Prevalence of Elevated and Hypertensive Blood Pressure^a^ Among US Children Aged 8 to 17 Years (N = 36,612,323)^b^, by Biennial Cycle, National Health and Nutrition Examination Survey (NHANES) 2011–2018

NHANES cycle	Elevated Blood Pressure Prevalence, % (95% CI)	Hypertensive Blood Pressure Prevalence %, (95% CI)
2011–2012	8.3 (6.4–10.7)	4.6 (3.5–6.1)
2013–2014	6.0 (4.6–8.0)	2.6 (1.7–3.8)
2015–2016	8.2 (6.6–10.3)	4.3 (2.9–6.3)
2017–2018	6.2 (4.2–9.3)	3.9 (2.9–5.3)

a Hypertensive and elevated blood pressure determined by 2017 American Academy of Pediatrics guidelines. Hypertensive: blood pressure percentile ≥95 or average blood pressure ≥130/80 mm Hg (whichever was lower) for children aged 8–12 years and ≥130/80 mm Hg for children aged ≥ 13 years. Elevated blood pressure: ≥90th percentile to <95th percentile or 120/<80 mm Hg to <95th percentile (whichever is lower) for children aged 8–12 years and 120/<80 to 129/<80 mm  Hg for children aged 13 to 17 years.

b Unweighted, N = 5,971.

**Elevated and hypertensive BP by child’s weight status**. Both elevated and hypertensive BP were more prevalent in children categorized as overweight or as having obesity compared with children of healthy weight. For elevated BP among overweight children, the prevalence difference was +4.3% (95% CI, 1.8%–6.8%). For children with obesity, the prevalence difference for elevated BP was +7.8% (95% CI, 5.7%–9.9%). For hypertensive BP, the prevalence difference for overweight children was +1.9% (95% CI, 0.3%–3.5%), and for children with obesity, the prevalence difference was +6.4% (95% CI, 4.3%– 8.6%) (Table 3). Children with BMIs within the range indicating obesity had a prevalence of hypertensive BP almost 4 times greater than those with healthy weight (8.6%; 95% CI, 6.9%–10.9%) versus 2.2% (95% CI, 1.7%–2.8%).

**Table 3:** Prevalence of Elevated and Hypertensive Blood Pressure^a^ by Sociodemographic Characteristics, US Children Aged 8 to 17 Years (N = 36,612,323)^b^, National Health and Nutrition Examination Survey (NHANES) 2011–2018

Characteristic	Elevated blood pressure			Hypertensive blood pressure		
	Prevalence, % (95% CI)	Crude Prevalence Difference (95% CI)	Prevalence Difference Adjusted for Overweight/Obesity, % (95% CI)	Prevalence, % (95% CI)	Crude Prevalence Difference (95% CI)	Prevalence Difference Adjusted for Overweight/Obesity, % (95% CI)
**BMI percentile^c^**						
Healthy weight, <85	4.9 (4.1 to 5.9)	Reference	NA	2.2 (1.7 to 2.8)	Reference	NA
Overweight, ≥85 to <95	9.2 (7.1 to 12.0)	4.3 (1.8 to 6.8)		4.1 (2.7 to 6.1)	1.9 (0.3 to 3.5)	
Obesity, ≥95	12.7 (10.7 to 15.1)	7.8 (5.7 to 9.9)		8.6 (6.9 to 10.9)	6.4 (4.3 to 8.6)	
**Age, y**						
8–9	5.9 (4.4 to 8.0)	Reference	Reference	6.0 (4.6 to 8.0)	Reference	Reference
10–12	4.3 (3.2 to 6.0)	−1.6 (−3.6 to 0.5)	−1.7 (−3.6 to 0.3)	4.0 (3.0 to 5.3)	−2.1 (−4.1 to 0.02)	−1.5 (−3.6 to 0.7)
13–15	7.0 (5.6 to 8.8)	1.1 (−1.2 to 3.4)	0.7 (−1.5 to 2.8)	2.0 (1.4 to 2.8)	−4.1 (−5.9 to −2.3)	−3.8 (−5.6 to −2.0)
16–17	12.8 (10.4 to 15.8)	6.9 (3.7 to 10.2)	6.3 (3.2 to 9.4)	4.5 (3.2 to 6.3)	−1.6 (−3.9 to 0.8)	−1.4 (−3.6 to 0.7)
**Sex**						
Female	4.9 (3.9 to 6.1)	Reference	Reference	3.0 (2.2 to 4.1)	Reference	Reference
Male	9.6 (8.1 to 11.2)	4.6 (2.8 to 6.5)	4.6 (2.7 to 6.4)	4.7 (3.7 to 5.9)	1.7 (0.2 to 3.2)	1.3 (−0.2 to 2.8)
**Race/ethnicity**						
Non-Latino White	6.3 (5.1 to 7.9)	Reference	Reference	3.2 (2.4 to 4.3)	Reference	Reference
Non-Latino Black	10.4 (8.8 to 12.1)	4.0 (2.1 to 5.9)	4.0 (2.2 to 5.8)	4.4 (3.3 to 5.8)	1.2 (−0.3 to 2.7)	0.5 (−0.8 to 1.9)
Mexican American	8.4 (6.8 to 10.5)	2.1 (−0.1 to 4.3)	1.6 (−0.5 to 3.7)	5.2 (3.9 to 6.8)	2.0 (0.1 to 3.9)	1.3 (−0.4 to 2.9)
Other Latino^d^	8.0 (6.0 to 10.6)	1.7 (−0.9 to 4.3)	1.7 (−0.7 to 4.1)	3.9 (2.4 to 6.2)	0.7 (−1.2 to 2.6)	0.3 (−1.5 to 2.0)
Non-Latino Asian^d^	4.6 (2.9 to 7.4)	−1.7 (−4.5 to 1.0)	−0.2 (−2.8 to 2.5)	4.3 (2.8 to 6.4)	1.1 (−0.7 to 2.9)	1.5 (−0.2 to 3.1)
Other^d^^,^ e	6.7 (4.2 to 10.7)	0.4 (−2.9 to 3.7)	0.2 (−2.7 to 3.1)	5.5 (3.5 to 8.6)	2.3 (−0.4 to 5.1)	1.9 (−0.6 to 4.4)
**Family education**						
≥College graduate	5.4 (4.0 to 7.4)	Reference	Reference	3.9 (2.7 to 5.5)	Reference	Reference
High school diploma/GED/some college	8.0 (6.8 to 9.4)	2.5 (0.6 to 4.5)	1.6 (−0.3 to 3.5)	3.8 (3.0 to 4.9)	−0.02 (−1.7 to 1.7)	−0.7 (−2.4 to 0.9)
<High school diploma	8.0 (6.5 to 9.9)	2.6 (0.4 to 4.8)	2.1 (0.0 to 4.3)	3.8 (2.8 to 5.2)	−0.06 (−1.8 to 1.7)	−0.5 (−2.3 to 1.3)
**Family income^f^**						
High	5.8 (4.2 to 7.9)	Reference	Reference	3.1 (2.1 to 4.5)	Reference	Reference
Medium	7.8 (6.5 to 9.2)	2.0 (−0.3 to 4.2)	1.3 (−1.1 to 3.6)	4.0 (3.1 to 5.2)	0.9 (−0.7 to 2.6)	0.4 (−0.9 to 1.7)
Low	8.4 (7.3 to 9.6)	2.2 (0.4 to 4.0)	1.4 (−0.4 to 3.2)	4.6 (3.6 to 5.8)	1.4 (−0.2 to 3.0)	0.6 (−0.8 to 2.1)

Abbreviation: NA, not applicable.

a Hypertensive and elevated blood pressure determined by 2017 American Academy of Pediatrics guidelines. Hypertensive: blood pressure percentile ≥95 or average blood pressure ≥130/80 mm Hg (whichever was lower) for children aged 8–12 years and ≥130/80 mm Hg for children aged ≥ 13 years. Elevated blood pressure: ≥90th percentile to <95th percentile or 120/<80 mm Hg to <95th percentile (whichever is lower) for children aged 8–12 years and 120/<80 to 129/<80 mm  Hg for children aged 13 to 17 years.

b Unweighted, N = 5,971.

c BMI (weight in kg/height in m^2^) as percentile according to the Centers for Disease Control and Prevention 2000 growth charts.

d Had fewer than 30 participants; therefore, did not meet NHANES reporting standards in the hypertensive category.

e Includes American Indian or Alaska Native, Native Hawaiian or Pacific Islander, and mixed race.

f Determined by family poverty income ratio; family income divided by Department of Health and Human Services poverty guidelines (specific to family size, year and sate of residence). High PIR = >3.5, medium PIR = ≥1.3 to <3.5, low PIR = <1.3.

**Sociodemographic differences in elevated BP prevalence**. Prevalence of elevated BP differed across sociodemographic groups. Prevalence was higher among males (9.6%; 95% CI, 8.1%–11.2%) than among females (4.9%; 95% CI, 3.9%–6.1%), and the difference remained significant after adjustment for body weight status (adjusted prevalence difference, +4.6%; 95% CI, 2.8%–6.5%) (Table 3). Prevalence was also greater among older children (16–17 y vs 8–9 y) before adjustment (crude prevalence difference +6.9%; 95% CI, 3.7%–10.2%) and after adjustment (adjusted prevalence difference, +6.3%; 95% CI, 3.2%–9.4%). Children of non-Latino Asian descent had the lowest crude prevalence of elevated BP (4.6%; 95% CI, 2.9%–7.4%), followed by non-Latino White children (6.3%; 95% CI, 5.1%–7.9%), whereas non-Latino Black children had significantly greater prevalence (10.4%; 95% CI, 8.8%–12.1%), with the crude prevalence difference +4.0% (95% CI, 2.1%–5.9%) (Table 3). After adjustment for weight status, these prevalence differences remained: +4.0 (95% CI, 2.2%–5.8%) among non-Latino Black children compared with non-Latino White children. Elevated BP also appeared to have an inverse relationship with socioeconomic status: the highest prevalence estimates were observed among children of low-income families (8.4%; 95% CI, 7.3%–9.6%) or from a household with parent/guardian educational attainment of less than a high school diploma (8.0%; 95% CI, 6.5%–9.9%) in unadjusted estimates. These socioeconomic differences were attenuated, and significance remained only when comparing those with the lowest parent/guardian education (<high school diploma) to those with the highest (college graduate or above) after adjustment for weight status (adjusted prevalence difference, +2.1%; 95% CI, 0%–4.3%).

**Sociodemographic differences in prevalence of hypertensive BP**. Prevalence of hypertensive BP also differed by sociodemographic groups as did crude and adjusted prevalence differences. Although the unadjusted prevalence estimates were higher among children in all racial/ethnic groups compared with non-Latino White children (unadjusted prevalence difference from +0.7% [95% CI, −1.2% to 2.6%] to +2.3 [95% CI −0.4% to 5.1%]), these differences were not significant (Table 3). The unadjusted prevalence of hypertensive BP was higher among male children (prevalence, +1.7%; 95% CI, 0.2%–3.2%) than female children, but this difference was no longer significant after adjustment for the differential distribution of weight status. The prevalence of hypertensive BP was lower among children aged 13 to 15 years compared with those aged 8 to 9 years (unadjusted prevalence difference, −4.1%; 95% CI, −5.9% to −2.3%), and these differences remained significant after adjustment for weight status (adjusted prevalence difference, −3.8%; 95% CI, −5.6% to −2.0%). No differences in hypertensive BP prevalence were seen across PIR levels or parent/guardian education levels.

## Discussion

Our study showed prevalence among children aged 8 to 17 years to be 7.2% for elevated BP and 3.8% for hypertensive BP according to 2017 AAP guidelines. Our findings also confirm the important relationship between body weight and BP among children aged 17 years or younger. Children who were classified as overweight or having obesity were more likely to have elevated or hypertensive BP than healthy-weight children. We identified associated sociodemographic differences and found that some, but not all, of these differences were attenuated after accounting for disparities in body weight (1,8,9). We found higher prevalence estimates of elevated BP in males, older children (16–17 y), non-Latino Black children, and children of lower socioeconomic status. After adjustment for weight status, elevated BP prevalence differences in age, sex, race/ethnicity, and parent/guardian education persisted in these groups. Hypertensive BP was highest among younger children (8–9 y), Mexican America children, and males.

The prevalence of elevated and hypertensive BP observed in our study is higher than previous estimates (7,8). These earlier estimates were based on previous guidelines where weight distribution skewed the normative tables resulting in higher BPs at lower percentiles and fewer children meeting the elevated and hypertensive percentiles (18). A previous study that used the 2017 AAP guidelines found a declining trend in hypertensive BP prevalence among children aged 8 to 17 years in NHANES data when comparing data collected in 2005–2008 with data collected in 2013–2016 (1). Focusing on more recent data and not aggregating biennial cycles, we found the prevalence of elevated and hypertensive BP to fluctuate between the study years of 2011 and 2018. However, overlapping confidence intervals suggest these differences were probably due to chance. The prevalence of elevated and hypertensive BPs was highest in the NHANES 2011–2012 cycle and lowest in 2013–2014. Past declining trends may have been misleading by not including the 2011–2012 cycle. Our prevalence estimate of 3.8% suggests that hypertensive BP among children remains an important public health issue and that the Healthy People 2020 goal of reducing this prevalence to 3.2% has thus far not been achieved (19).

Our study confirmed results of previous studies that showed overweight and obesity to be major risk factors for high BP in children (2–5,7,9,20) and supports changes in the AAP guidelines to the use of BP tables based on children of healthy body weight. In our study, adjustment for weight resulted in the attenuation of prevalence differences in elevated and hypertensive BP across the sociodemographic groups examined, emphasizing the influence of weight on observed disparities in BP. Thus, future studies that examine sociodemographic differences in children’s BP levels need to adjust for the child’s weight in further stratified or multivariable adjusted regression analyses to more systematically examine differences across any strata under study.

Consistent with the published literature, our findings suggest that in unadjusted estimates male children, children with parent/guardian with lower levels of education, and children from families with low income levels experienced a greater burden of cardiovascular risk because of disproportionate rates of unhealthy body weight (21). Sex differences in physiologic parameters, such as total cholesterol levels, and health behaviors, such as physical activity levels, have previously been highlighted in relation to childhood obesity and could contribute to the higher unadjusted prevalence of hypertensive BP observed among males (21). Disparities in the built environment, which affect patterns of physical activity, and access to healthy foods at affordable prices are acknowledged risk factors for children of low socioeconomic status who are overweight and could contribute to the higher unadjusted prevalence of elevated BP observed in children with low levels of parent/guardian education or income (22,23). Thus, through various weight-related pathways and mediators, weight-related disparities may contribute to disparities in unadjusted prevalence of BP levels across the sociodemographic factors of sex, education, and family income.

The crude racial/ethnic prevalence differences detected in our study underscore the disproportionate burden of elevated BP and unhealthy weight in non-Latino Black communities (24,25). Numerous factors across socioecological levels have been noted to contribute to disproportionate obesity prevalence across racial/ethnic groups (24,25). Here again, we see that factors contributing to weight disparities may also contribute to BP-related disparities (23). Weight-related risk factors can be systematic and range from health care access to safety and opportunity (26). Beyond describing their existence, more action needs to be taken to disentangle and prevent the factors contributing to these disparities to achieve health equity.

In our study, racial/ethnic disparities in prevalence of elevated and hypertensive BP remained after adjusting for weight status. This indicates that factors other than body weight contribute to racial/ethnic disparities in children’s BP and that other pathways to less than optimal BP levels may begin in childhood. One such pathway is psychosocial stress, which has been extensively studied in adult populations (27). Empirical investigation of pathways (obesity-related and other) to racial/ethnic disparities in elevated BP prevalence is warranted as are interventional and policy-based efforts designed to narrow these differences and lower children’s risk of subsequent cardiovascular disease. Weight disparities did not fully explain observed differences in elevated BP prevalence by sex in our study. In adult populations, sex-related BP differences are well established (28), and our findings suggest that the pathways to these sex-related BP differences may begin in childhood.

The differences we found in prevalence estimates of elevated and hypertensive BP in relation to age may be due in part to increased BP variability among young children (29) and in the use of percentile-based definitions for children aged 8 to 12 years compared with static cutoffs for children aged 13 to 17 years (30). Additionally, prevalence differences detected across age groups could be due to changes in BP associated with puberty and to the intersection of these changes with age, sex, and race/ethnicity. Further understanding is needed about how levels of BP disorders differ, and long-term follow-up data on BP levels among children are needed.

Our study highlights opportunities for reduction of elevated and hypertensive BP levels among US children. Efforts focusing on increased equity in access to care through policy changes to combat obesity in racially/ethnically and socioeconomically diverse populations should be expanded. Specific focus and efforts directed at systematic change to improve social determinants of health are also needed. Efforts to understand the causes of racial/ethnic and socioeconomic disparities and to reduce them could have short- and long-term benefits through improvements in children’s health and long-term prevention into adulthood (31). Given the well-known tracking of BP into the adult years and the strong association between elevated BP and cardiovascular and other chronic diseases, particular focus on preventing the large number of males with elevated BP from progressing to hypertension is warranted (32). Further research and risk reduction approaches should be directed to expanding BP screening in national samples of young children to improve our understanding of childhood hypertensive BP and reduce the risk of chronic diseases associated with hypertension later in life. Clinicians should be aware of socioeconomic disparities and the role of overweight highlighted in our study.

Strengths of the present study come from its use of contemporary nationally representative data and current BP screening guidelines. Although assessing subgroup differences in children’s elevated and hypertensive BP may be difficult because of low case counts, we were able to combine the 4 most recent NHANES data cycles to obtain contemporary estimates across sociodemographic groups. The data analyzed in our study were collected by trained professionals who used standardized methods under controlled conditions and with quality control measures. This is important because collecting accurate BP measurements among children can be challenging (5).

Our study also has limitations. Despite the strengths inherent in the use of NHANES data, the study was limited by the data collected in that survey. Although declining response rates are of concern, NHANES has taken steps to mitigate the potential for nonresponse bias (13). Blood pressure measurements were limited to a single occasion rather than a series on 3 occasions, as is necessary for clinical diagnosis. However, previous childhood hypertension studies also used readings from a single occasion, including those providing national prevalence estimates (1,5). No single measure accurately reflects socioeconomic status, and we were unable to evaluate food insecurity as a marker of socioeconomic status, or low birthweight as a potential confounder, because NHANES assesses these measures only in children aged 16 years or older. Data on other important, potentially confounding variables, including family history of hypertension, chronic kidney disease, and chronic sleep disturbance were not available.

Elevated and hypertensive BP affects US children disproportionately in various sociodemographic groups, and body weight influences these health disparities. The burden of this cardiovascular risk is higher in children who are male, non-Latino Black, or of low socioeconomic status. Age, sex, and race/ethnicity may influence BP independently of weight status. Efforts are needed to better understand and intervene on the mechanisms through which these factors interact with BP in children. Obesity and hypertension are preventable disorders that potentially cause lifelong harm. Continued and amplified efforts are needed related to elevated and hypertensive BP among children aimed at lowering the prevalence, decreasing disparities, and ultimately achieving health equity.
